# Let Students Work: Analysis of the Role of Differing Facilitation on Student Engagement in a Large Stadium-Style Lecture Hall

**DOI:** 10.1021/acs.jchemed.3c00750

**Published:** 2023-11-01

**Authors:** Nicole E. States, Carson Lovig, Karsten Martin, Hannah T. Nennig, Renée S. Cole

**Affiliations:** Department of Chemistry, University of Iowa, Iowa City, Iowa 52242, United States

**Keywords:** First-Year Undergraduate, Chemical Education Research, Curriculum, Constructivism, Collaborative/Cooperative Learning, Student-Centered Learning

## Abstract

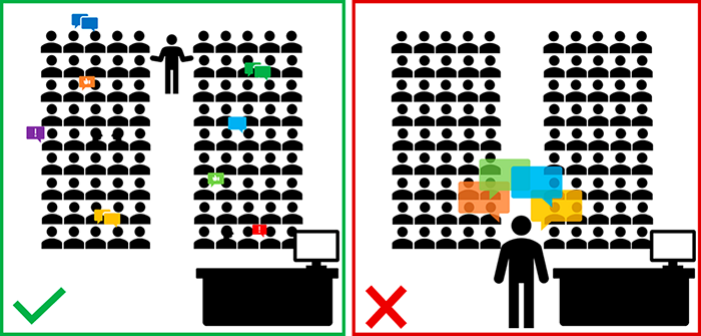

The classroom environment is shaped by factors such as facilitation style, curricular design, and classroom layout. These factors are all inputs into student framing of the classroom environment and affect a student’s comfort interacting within it. Promoting student discourse in active learning environments provides students the opportunity to explain their thinking and develop their understanding of natural phenomena. However, successfully implementing these practices in large lecture environments is often difficult. Undergraduate introductory chemistry lectures were investigated to identify the effects that instructional practices had on student engagement. Instructor facilitation, question level, and student interactions were analyzed and compared to provide insights into what instructional practices may promote or hinder student engagement in a large enrollment course. Overall instructors were positioning themselves as an authority on knowledge in the classroom by leading questions authoritatively like instructor-focused didactic lecturing that led to a decrease in student engagement. These results highlight the complexity of the classroom ecosystem related to student interactions and the role that facilitation plays in social and cognitive engagement.

## Introduction

### Facilitation Affects the Ways Students Engage in a Classroom

A focus of chemistry education research has been on developing and investigating the effects of evidence-based instructional practices (EBIPs)^1−6^ and the adoption of such practices in science, technology, engineering, and mathematics (STEM) classrooms.^7−11^ Despite the abundance of work on evidence-based instructional practices, simply using an EBIP does not guarantee positive student outcomes.^12^ Stains and Vickrey highlighted that most research investigating the impact of EBIPs often assumed that implementation was as intended by the developers.^13^ However, instructor facilitation shapes the classroom environment and lays the foundation for student interactions.^14,15^ Instructors’ actions and the classroom norms they set may impact a student’s comfort and expectations for interacting in that setting.

Research has highlighted the importance of facilitation in STEM classrooms, but work conducted in this area occurred in small classroom settings.^14,16−23^ The effect of EBIPs has been shown to be dependent on how the practices are specifically implemented by an instructor.^10,24^ Aina, Sunday, and Ayinde proposed a conceptual framework^25^ that highlighted key variables such as assessment, instructional delivery, personal qualities of the teacher, and the learning environment that play a role in teaching effectiveness. Regarding the classroom learning environment, when attempting to apply EBIPs in traditional, large enrollment classrooms with fixed seating, practices are often not able to be implemented fully in their intended way, so there is a need to investigate the facilitation of common instructional practices within these settings.

### The Classroom Setting Affects the Way Instructors Facilitate Learning

The classroom layout can have an impact on the implementation of research-based instructional practices. For example, classrooms with stadium-style seating make it difficult to keep track of what students are doing.^10,14,23^ Brooks^26^ completed a study that examined the influence of classroom layout on student success in an introductory biology course. This study used the same instructor, curriculum, time of day the class was offered, and exams for a course offered in a traditional stadium-style classroom and a reformed active learning classroom with round tables designed to promote group work. It was found that students in the active learning classroom outperformed grade expectations and the students in the traditional classroom.^26^ A follow-up study showed similar results in that students in the active learning classrooms outperformed grade expectations.^27^ Because traditional classrooms with fixed seating place emphasis on the instructor providing information over students engaging in group work, it can be more difficult for students to prioritize collaborative interactions.

Despite the research highlighting the benefits of active learning classrooms over traditional classrooms for STEM courses, it is not feasible for many institutions to make changes to physical spaces due to insufficient resources. With the ongoing need to incorporate more inclusive teaching practices, traditional classroom barriers continue to limit instructor motivation to incorporate active learning practices.^9,11,28^ These barriers can lead to a deficit mindset positing that students need to survive the current system because there is no way to change the institution.^29^ Questions delivered using student response systems, commonly referred to as “clicker questions,” have been shown to be a promising way to integrate active learning into stadium-style classrooms because of their low cost and ease of use,^4,30^ but the implementation of clicker questions is key to their success.^31,32^ Given the prevalence of stadium-style classrooms that rely solely on clicker questions for student engagement,^28^ it is crucial to assess the efficacy of facilitation strategies that incorporate these questions in such settings. This work is focused on identifying instructional practices that hinder or promote student engagement in large lecture settings.

## Theoretical Framework

Lawson and Lawson^33^ proposed a cyclic transactional framework for student engagement that informed the collection and analysis of data and interpretation of the results for this study. The framework explains that when observing student acts of engagement, there are conditions for engagement as well as dispositions and drivers that promote or deter student engagement. The key difference between conditions for engagement versus dispositions and drivers for engagement is that conditions are external to the students, such as the classroom layout, whereas dispositions and drivers are internal motivations from the student. The acts of engagement that students choose to take leads to benefits and competencies that cyclically provide feedback on students’ conditions as well as their dispositions and drivers for engagement that affect how they choose to engage in the next question. In this paper, we focus on uncovering the conditions for engagement that differing facilitation strategies had on students’ acts of engagement across two semesters in a large enrollment introductory chemistry classroom.

## Methods

### Setting

This study took place at a large research-intensive university in the United States across two successive semesters of a first-semester, large-enrollment, introductory chemistry course. Students in this course attended three lectures, one discussion, and one laboratory/case study session each week. There were three lecture sections for the course, although only one section was analyzed in this study. The lecture section studied met three times a week for 50 min in the same traditional, stadium-style lecture hall with 390 seat capacity and approximately 250 students enrolled. Each semester of the course was team-taught by three instructors who rotated in the classroom across semester topics.

### Participants

Students in this classroom were not required to sit in groups to work on the student response system questions, so groups for analysis were chosen based on the observational data to identify students who interacted and sat together consistently before the first exam. During semester one, two student groups were selected, each consisting of three members. During semester two, four student groups with 2–4 students were selected. At any given time during the semester, there was one faculty instructor and three graduate teaching assistants (GTAs) in the room. The instructors for this course were given pseudonyms and taught at alternating intervals across each semester. Instructors Green, Blue, and Yellow taught during semester one and Instructors Purple, Orange, and Pink taught during semester two. The schedule of their instruction is shown in Table 1.

**Table 1:** Number of Lectures Observed and Order of Instructors across Each Semester

Semester One			
Instructor	Green	Blue	Yellow
Number of observed lectures	5	9	10

Semester Two						
Instructor	Orange	Pink	Purple	Pink	Orange	Purple
Number of observed lectures	4	4	4	7	5	5

During the classroom lecture, each instructor lectured from the front of the room using PowerPoint slides and periodically asked questions to be answered using a student response system after discussion with classmates. Each instructor asked at least one question per class period, with a maximum of nine questions. The questions were displayed on both a PowerPoint slide and on the student response system screen. Variable amounts of time were allotted for each question, with the shortest being 45 s and the longest being 7 min. Students submitted answers to questions individually, and bonus points for participation were awarded if students answered most questions throughout the semester. While students were working on questions, GTAs would stand in the aisles to answer any student questions. Instructor Green from semester one and all the instructors in semester two would circulate the room, observing student progress, checking in with students, and answering questions. Instructors Blue and Yellow from semester one, however, tended to stay at the front of the room waiting for students to reach out to them with questions. When students were not answering student response system questions, they were listening to the lecture and sometimes taking notes or annotating lecture slides.

### Data Collection

Data collection for this study included observational notes by the first author and video and audio recordings of the classroom during regular lecture periods. Observational notes were collected to provide data about classroom norms and the implementation of questions. Video cameras were placed at the front of the room to capture classroom interactions and interactions with the instructor. Audio interactions of students completing clicker questions were collected using an audio recorder. IRB-approved informed consent documents were signed by each instructor and small group member before the recording took place.

## Data Analysis

### Analysis of Instructor Facilitation

#### Question Coding

Questions administered to students were coded according to the cognitive level of questions as described by Marzano’s taxonomy.^34^ Marzano’s taxonomy allowed us to classify questions based on hierarchical cognitive processes expected for students to answer the questions, providing information about how questions were designed to promote cognitive engagement. A stratified sample of 55 out of 182 questions coded that encompassed a representative sample across all semesters and instructors was selected for analysis by a second researcher to establish interrater reliability. This sample represented 30% of all coded data. Cohen’s kappa values were found to be 0.71 for question level, showing moderate to strong reliability between coders. The coded data was trustworthy to be used for further analysis according to the guidance found in McHugh’s Interrater reliability: the kappa statistic.^35^ Disagreements were negotiated, and the remaining coding was completed by authors one and three.

#### Video Coding

Video recordings were trimmed to only include portions of the video when instructors were delivering questions for student group work. Video data were qualitatively coded using the coding software MAXQDA.^36^ Videos were coded with three levels of specificity. The first level denoted the phases of the question delivery to students. The codes in this category were introduction, during, and closing. Descriptions of the codes can be found in Table 2.

**Table 2:** Definitions for the Phases of Question Delivery Codes

Code	Definition
Introduction of Question	The instructor is initiating the question(s) that the students are supposed to answer for a given time period.
During Question	Instructor actions occurring during the time allotted for students to work on the introduced question(s).
Closing of Question	The instructor is closing the question that students were supposed to complete.

The second level of coding was categorizing the communicative approach, which describes the communication style of the instructor. One communicative approach code was applied for each phase of the question delivery. The communicative approach codes were developed by Mortimer and Scott,^37^ and their definitions can be found in Table 3.

**Table 3:** Adapted Definitions of Communicative Approach Codes from Mortimer and Scott^37^

Communicative Approach	Definition
Interactive Dialogical	Teachers and students consider a range of ideas during the discussion. If the influence of student ideas is high, they pose genuine questions as they explore and work on different points of view. If the influence that students’ ideas have is low, the different ideas are simply made available during discussion.
Noninteractive Dialogical	The teacher revisits and summarizes different points of view, either simply listing them or exploring similarities and differences.
Interactive Authoritative	The teacher focuses on one specific point of view and leads students through a question-and-answer routine with the aim of establishing and consolidating that point of view.
Noninteractive Authoritative	The teacher presents a specific point of view.

The last level of coding was the focus of instructor interactions. These codes were developed by authors one, two, and four through an open coding process as described by Merriam and Tisdell.^38^ Three code categories were developed from the iterative open coding: managing, questioning, and relaying. Managing codes were applied when the instructor gave students information that did not relate to the question answers. Questioning codes were applied when an instructor inquired about student(s) knowledge. Relaying codes were applied when an instructor provided content information that relates to the question. Code definitions for instructor interaction moves can be found in Tables 4, 5, and 6.

**Table 4:** Definitions for Managing Instructor Interaction Moves

Code	Definition
Announcing Question Period	The instructor opens the question-answering period for the specific prompt(s).
Call on Student	The instructor opens the floor for a particular student to speak.
Classroom Management	The instructor brings students’ attention back to the question.
Closing Class	The instructor dismisses the class.
Closing Question Period	The instructor closes the question answering period.
Course Reminders	The instructor provides information that is related to the course but does not directly impact the question(s).
Encouragement	The instructor motivates the students.
Encouraging Collaboration	The instructor encourages students to work with their group or individuals around them, or ask for instructional aid when needed.
Opening Class Period	The instructor opens the class period for students to work on problems.
Overview	The instructor gives a conceptual summary of what the students will be working on for that day’s activities or specific prompt(s).
Giving Directions	The instructor provides students with information regarding the class procedure(s) and/or prompt(s) that does not directly aid in the students’ solving process.
Reading Prompt	The instructor presents the prompt(s).
Reconsider Answer	The instructor asks the students to review their answer.
Time Information	The instructor provides information related to the duration of the question answering period or the timing of the question.
Study	The instructor encourages students to practice a concept and/or skill outside of the class period.

**Table 5:** Definitions for Questioning Instructor Interaction Moves

Code	Definition
Asks Content Question (Answered)	The instructor asks the students a question that is related to course content and it is answered.
Asks Content Question (Unanswered)	The instructor asks the students a question that is related to course content and it is not answered.
Asks for Questions	The instructor asks student(s) if they have any questions.
Asks for Whole Class Response (Content)	The instructor asks the whole class a question that is related to the course content.
Asks for Whole Class Response (Noncontent)	The instructor asks the whole class a question that is not related to the course content.
Asks Noncontent Question (Answered)	The instructor asks the students a question that is not related to course content and it is answered.
Asks Noncontent Question (Unanswered)	The instructor asks the students a question that is not related to the course content and it is not answered.
Cold Call Asks Question	The instructor asks a question to a specific group or individual.
Evaluate Progress	The instructor asks a question to assess how far students have progressed in the question-answering process.
Rhetorical Asks for Questions	The instructor prompts students with questions but does not allow time for them to respond.

**Table 6:** Definitions for Relaying Instructor Interaction Moves

Interaction	Definition
Answer Assessment	The instructor provides information related to the outcome of student responses.
Answer Student Question	The instructor responds to a student’s question.
Explains Answer	The instructor explains how to complete part or all of the question
Gives Analogy	The instructor gives an example that relates concepts elicited in the prompt that are not directly from the prompt.
Giving Hint	The instructor gives information to the students that will aid, without answering, the question-completion process.
Provides Question Answer	The instructor gives the answer or part of the answer to the question(s).
Responds to Student Answer	The instructor restates or briefly affirms students’ answers.

A stratified sample of 20 question deliveries out of the total 181 question deliveries that encompassed all semesters and instructors was selected for analysis by a second researcher. This sample represented 11% of all the coded data. Pairwise coding was completed on this subset of data by authors one, two and four and Cohen’s kappa statistic values were calculated (Table 7).

**Table 7:** Cohen’s Kappa Values of the Pairwise Coding between Authors One, Two, and Four

Coders	Author 1 and Author 2	Author 1 and Author 4	Author 2 and Author 4
Cohen’s Kappa	0.73	0.66	0.65

All values fell within the moderate range of agreement (0.60–0.80), and it was deemed moderate levels of agreement were sufficient for the study given the complex nature of interactions observed from audio-only data. The coded data was trustworthy to be used for further analysis according to the guidance found in McHugh’s Interrater reliability: the kappa statistic.^35^ Disagreements were negotiated, and the remaining coding was completed by authors one and two.

### Analysis of Student Interactions

The social processing and knowledge dynamic codes used to analyze student interactions in this study are discussed in a previous article.^39^ Social processing describes how students in groups interact with each other during a question-answering period. Knowledge dynamic describes the way that students interact with content knowledge to answer the question. These two categories were chosen for analysis because they provide insights into the social and cognitive engagement of student interactions while completing in-class questions. One additional code labeled “Leader” was added to the social processing scheme used in the previous study^39^ because it was observed that many interactions taking place with only one student talking were not because the student would reject the ideas of others, but that no other students would try to collaborate. Code definitions for social processing and knowledge dynamic used in this study can be found in the Supporting Information (Table S1 and Table S2).

A stratified sample of 118 out of 588 total student interactions recorded that encompassed a representative sample across all semesters, instructors, and groups was selected for analysis by a second researcher. This sample represented 20% of all the coded data. Cohen’s kappa values were found to be 0.69 for social processing and 0.80 for knowledge dynamic, showing moderate to strong reliability between coders. It was deemed that the moderate levels of agreement of social processing were sufficient for the study given the complex nature of student interactions observed from audio-only data. The coded data was trustworthy to be used for further analysis according to the guidance found in McHugh’s Interrater reliability: the kappa statistic.^35^ The disagreements were negotiated and the remaining coding was completed by authors one and three. Student interactions from semester one were transcribed verbatim to facilitate more detailed analysis of conversational turns. A conversational turn was the utterances spoken by one student before another student spoke.

## Results and Discussion

The goal of this work was to evaluate the effects of facilitation strategies of different instructors on student engagement in a large enrollment lecture hall. Student group and facilitation audio were analyzed during “clicker” questions across the semester to characterize the trends seen for different instructional practices and student engagement. The Results and Discussion section describes the qualitative trends in facilitation and the observed variance in student interactions.

### Student Social and Cognitive Engagement Varied across Instructors

**Figure 1:**
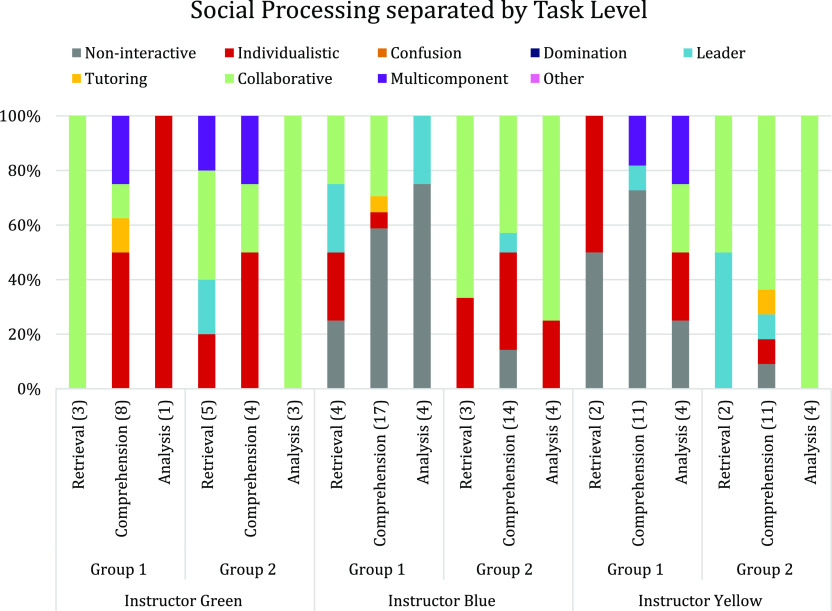
Percentages of each type of social processing across student groups during semester one separated by question level and instructor.

Student interactions were observed across semesters one and two and analyzed for the modes of social and cognitive engagement. Student engagement across question levels was analyzed because previous research has shown that the cognitive level of questions influences students’ social and cognitive engagement.^39^ Retrieval, comprehension, and analysis level questions were asked in both semesters, while metacognition questions were also asked by Instructor Pink during semester two. While different instructors asked a different number of questions, the proportions of retrieval, comprehension, and analysis level questions were similar across instructors within each semester. Percentages of each question level observed across semesters one and two can be found in the Supporting Information (Figures S1 and S2). We investigated if there were changes in engagement based on facilitation, including the question level of questions asked by each of the instructors. The percentage of each mode of social engagement across semester one separated by question level, instructor, and group can be seen in Figure 1. Plots demonstrating the social processing trends separated by both question level and group for each instructor in semester two can be found in the Supporting Information (Figures S3–S5).

The social dynamics of group one and group two differed across the semester. Group one was less interactive, with most of their interactions across all question levels being noninteractive or individualistic. In contrast, group two was more interactive, primarily engaging collaboratively. When looking at the trends of social engagement separated by question level, there were mixed results on whether increasing the cognitive complexity of the question promoted more collaborative engagement. For group one, increasing the question level had the opposite effect during the time that both Instructor Green and Instructor Blue taught, as an increase in either individualistic or noninteractive social processing was seen. However, during the time Instructor Yellow taught, group one did engage collaboratively when answering analysis level questions. Group two’s interactions, however, matched trends reported previously^39^ with an increase in collaborative engagement as the question level increased. Variations in group dynamics with question level were also observed for the groups in semester two. Given the differences observed across the groups, question level did not appear to be a consistent driver for collaborative social engagement in this setting.

In addition to analyzing the impact of increasing the question level on social engagement, we looked at the trends of social processing across the semester as different instructors taught. At the beginning of the semester, group one was primarily engaging individualistically, but when Instructor Blue took over, the group became mostly noninteractive. This noninteractive mode continued throughout Instructor Yellow’s instruction as well. This indicates that there was a shift in the acts of engagement displayed by group one with a change of instructor. During Instructor Blue’s teaching, group two also increased in individualistic and noninteractive behaviors. However, when Instructor Yellow taught after Instructor Blue, group two recovered and became more social and interactive while completing tasks. In both semesters one and two, it was observed that the types of social processing differed across instructors.

**Figure 2:**
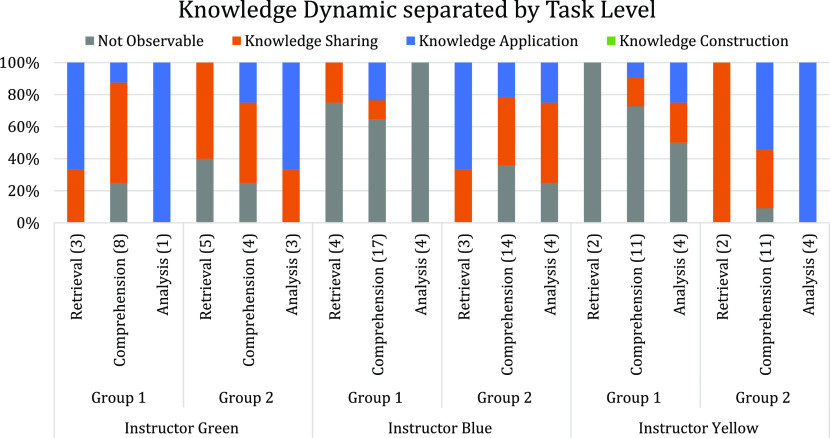
Percentages of each knowledge dynamic across student groups during semester one separated by question level.

Next, the authors analyzed the knowledge dynamics observed during group interactions to see if there were differences in knowledge discourse used by the groups. The percentage of each knowledge dynamic seen across semester one was separated by question level, instructor, and group, and can be seen in Figure 2. Similar plots showing the knowledge dynamic separated by both question level and instructor for semester two can be found in the Supporting Information (Figures S6–S8).

As was observed in their social engagement, student groups interacted differently with each other in their knowledge discourse across the semester. No knowledge construction was observed for either group, which means that the students did not collaboratively build on each other’s ideas to construct new understandings as they completed the questions asked in class. The noninteractive nature of group one limited the ability to characterize the way they engaged with knowledge. When they did interact, the primary mode was knowledge sharing with some knowledge application. In contrast, group two was more consistent in exchanging ideas across the semester as well as engaging in more knowledge application. When looking at the effect of question level on knowledge discourse, the relationship between the cognitive complexity of the question and the nature of knowledge discourse also depended on which instructor was present. While Instructor Green was teaching, most of group one’s discourse was sharing or applying knowledge, but there was not a consistent increase in knowledge management as the question level increased. There was a distinct shift in the acts of knowledge use displayed by group one when Instructor Blue took over instruction, with group one mostly not interacting with knowledge as a group. When Instructor Yellow began teaching, group one was still primarily not interactively engaged, but knowledge sharing and applying knowledge was observed in greater amounts as the question level increased. This highlights the inconsistencies seen in knowledge discourse across instructors as the trends in group one’s knowledge use only match the previous literature^39^ when Instructor Yellow was teaching.

Group two also engaged with knowledge differently based on question level across instructors. While Instructor Green was teaching, group two interacted as seen in prior work^39^ with an increase in applying knowledge seen as question level increased. However, during the time Instructor Blue taught, there was an increase in times where student groups did not interact with knowledge for both comprehension and analysis level questions. Additionally, when students were engaging with knowledge, group two did not apply knowledge more often as question level increased. When Instructor Yellow began teaching, the trend became consistent again with previous work, showing an increase in applying knowledge as question level was increased. With both groups, we saw inconsistent results on whether increasing the question level would lead to higher levels of knowledge management. It was also seen that the trends of knowledge dynamic observed differed across instructors in semester two.

### Facilitation of Questions Was Inconsistent

To characterize the facilitation strategies used to deliver clicker questions, instructor interactions were coded for their communicative approach and focus of the interaction for each phase of question delivery. First, we looked at the communicative approach. The introduction of questions and during question facilitation were all led noninteractive authoritatively for each instructor, and all during interaction interruptions were to the whole class. There were differences seen in the closing of questions, so we analyzed the communicative approach of the closing of a question in more detail. Frequencies of each communicative approach for closing questions can be found in Table 8.

**Table 8:** Frequency and Percentage of the Communicative Approach of Closing Question Codes for Each Instructor in Semester One

Instructor	Noninteractive Authoritative	Interactive Authoritative	Noninteractive Dialogical	Interactive Dialogical
Green	3 (20%)	12 (80%)	0 (0%)	0 (0%)
Blue	4 (15%)	22 (81%)	0 (0%)	1 (4%)
Yellow	15 (88%)	2 (12%)	0 (0%)	0 (0%)

Primarily authoritative approaches were used, with only one instance of an interactive dialogical communicative approach used by Instructor Blue. Instructor Yellow did not interact with students while explaining answers, with 88% of their communicative approaches for the closing of questions being noninteractive authoritative. This lack of interaction may have caused the students to become less interactive as they were not expected to share their thoughts during the closing. This may have led to less interactive social and cognitive engagement observed among the groups because the conditions for engagement did not promote interactive acts as it did with during the time Instructor Green taught.

**Figure 3:**
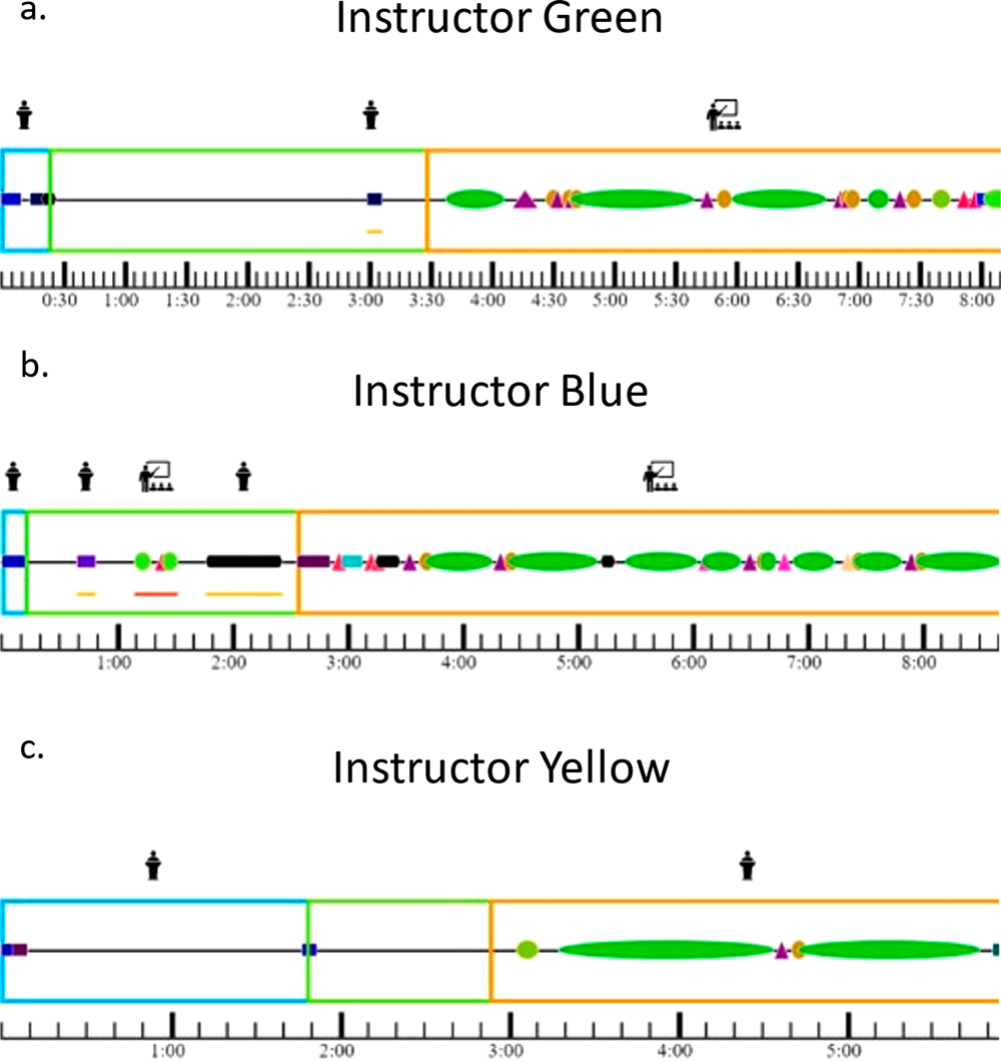
Facilitation patterns of Instructor Green (a), Instructor Blue (b), and Instructor Yellow (c). Blue boxes indicate the introduction of a question, green boxes are during a question, and orange boxes are the closing of question segments. Managing codes are squares, questioning codes are triangles, and relaying codes are circles. Individual colors for codes denote specific codes within each content of instructor interaction moves. A full key can be found in Table S3.

Instructor Green and Instructor Blue engaged students more actively while explaining questions, with both having about 80% of their communicative approaches for the closing of questions being interactive and authoritative. This would reasonably lead to the expectation that Instructor Blue and Instructor Green should have similar amounts of social and cognitive engagement from students, but we saw from above that there were distinct drops in the engagement of groups one and two during the time Instructor Blue taught. From this, it seems that the communicative approach and expectation for sharing out thoughts during the closing of a question may not have been enough of a motivator for engagement in a large enrollment classroom. This mismatch in expected outcomes prompted us to investigate the facilitation strategies used by each instructor in semester one more closely. Exemplars of facilitation patterns for each instructor are shown in Figure 3.

Looking at Figure 3, we can see that each of the instructors spent very little time introducing questions. Most of these sections were filled with the instructors announcing to students they were going to be working on a clicker problem in groups. The closing of questions were the longest sections for all instructors, meaning that the instructors spent more time explaining the answer than they allotted for students to solve the problem. This places a focus on the instructor as the authoritative keeper of knowledge in a similar way to didactic lecturing. This may have led to less social and cognitive engagement across groups due to the promotion of instructor-focused conditions for engagement.

The closing of questions for Instructor Yellow (Figure 3c) was primarily relaying information with long stretches of explaining the answer directly to the students in a didactic style, illustrating the noninteractive authoritative way Instructor Yellow closed questions. Instructor Blue’s (Figure 3b) and Instructor Green’s (Figure 3a) profiles show they engaged in acts that both managed the classroom and asked questions to the students in alignment with their primarily interactive communication style while closing questions. Another distinct difference observed in instruction is how instructors manage the time when students are working on clicker questions. Instructor Green (Figure 3a) and Instructor Yellow (Figure 3c) did not interrupt students very much when students were working on questions, but Instructor Blue (Figure 3b) regularly interrupted students while they worked in groups. This may have placed a larger focus on the instructor within the classroom than when the other two instructors were teaching that led to the drop in social and cognitive engagement observed during their instruction. The number of interruptions by Instructor Blue during time that was supposed to be used for student groups to work together may have shifted student’s dispositions for engagement as it became normal to be listening to the instructor during that time instead of working collaboratively.

We continued this analysis of communicative approaches and facilitation patterns to semester two to further characterize the role of facilitation on student interactions. For each instructor, most question introductions and during-question facilitation were done noninteractively and authoritatively, and any interruptions during interactions were directed to the entire class. Only two introductions took place interactively and authoritatively, and only by Instructor Pink, making up just 2% of the introductions. The small number of introductions from a lone professor done interactively was likely not enough to set up any consistent conditions for engagement. Similar to semester one, there were differences seen in how instructors closed questions, so we analyzed the communicative approach for this aspect of facilitation in more detail. Frequencies of each communicative approach to the closing of questions in semester two can be found in Table 9.

**Table 9:** Frequency and Percentage of Communicative Approach While Closing Questions for Each Instructor in Semester Two

Instructor	Noninteractive Authoritative	Interactive Authoritative	Noninteractive Dialogical	Interactive Dialogical	No Closing
Purple	8 (47%)	9 (53%)	0 (0%)	0 (0%)	0 (0%)
Orange	24 (68%)	11 (32%)	0 (0%)	0 (0%)	0 (0%)
Pink	61 (88%)	2 (2%)	2 (2%)	0 (0%)	4 (6%)

**Figure 4:**
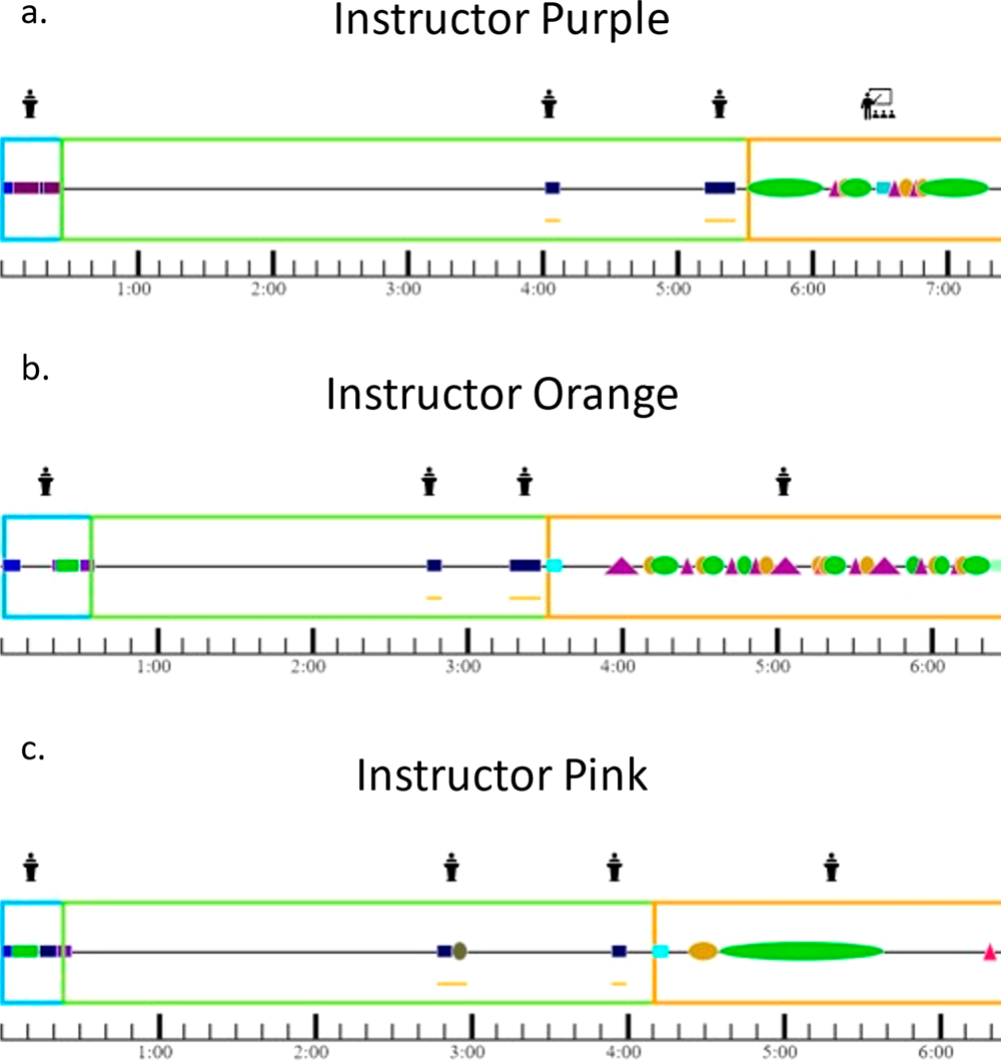
Facilitation patterns of Instructor Purple (a), Instructor Orange (b), and Instructor Pink (c). Blue boxes indicate the introduction of a question, green boxes are during a question, and orange boxes are the closing of question segments. Managing codes are squares, questioning codes are triangles, and relaying codes are circles. Individual colors for codes denote specific codes within each content of instructor interaction moves. A full key can be found in Table S3.

From the table, we see that primarily authoritative approaches were used, with only two noninteractive dialogical and four instances of no closing seen with Instructor Pink. Both Instructor Orange and Instructor Pink primarily closed questions using a noninteractive authoritative approach. To take a closer look at facilitation patterns to examine differences, we mapped the facilitation moves. Exemplars of facilitation from each instructor can be seen in Figure 4.

During semester two, each of the instructors taught at different times throughout the semester, but their profiles were similar each time they taught. The introduction of question sections was the shortest phase of question delivery and still primarily consisted of announcing the questions and reading the prompt. Different in semester two was that a larger proportion of time was allotted for students to answer the questions than for instructors to close the question. This means that the instructors during semester two were placing a larger focus on students working on problems in their groups than on the instructors explaining the answer. This established a norm across all instructors that students would have sufficient time allotted to work together on problems. This focus on allowing students time to work may have led to the larger amount of collaboration and higher levels of cognitive engagement seen in semester two as the conditions for engagement focused more on the students than in semester one.

The content moves observed during the question closure of Instructor Pink (Figure 4c) and Instructor Orange (Figure 4b) were primarily relaying information, which is consistent with the pattern seen in semester one. Instructor Purple primarily closed questions interactively, asking questions and relaying information as illustrated in the plot (Figure 4a). The conditions for engagement were set with the expectation, under Instructor Purple’s guidance, that students may be asked to share their thoughts in class. This may have been the reason for the higher levels of social engagement observed during their instruction. Instructor Pink had a much larger percentage of their communicative approaches being noninteractive while closing questions than Instructor Orange. The additional focus on the instructor not requesting student thoughts during the question closure may have set the condition for engagement to be centered around the instructor instead of the student, resulting in the lowest levels of social and cognitive engagement during the time Instructor Pink taught.

### Student Conversation Diminished When Interrupted

**Figure 5:**
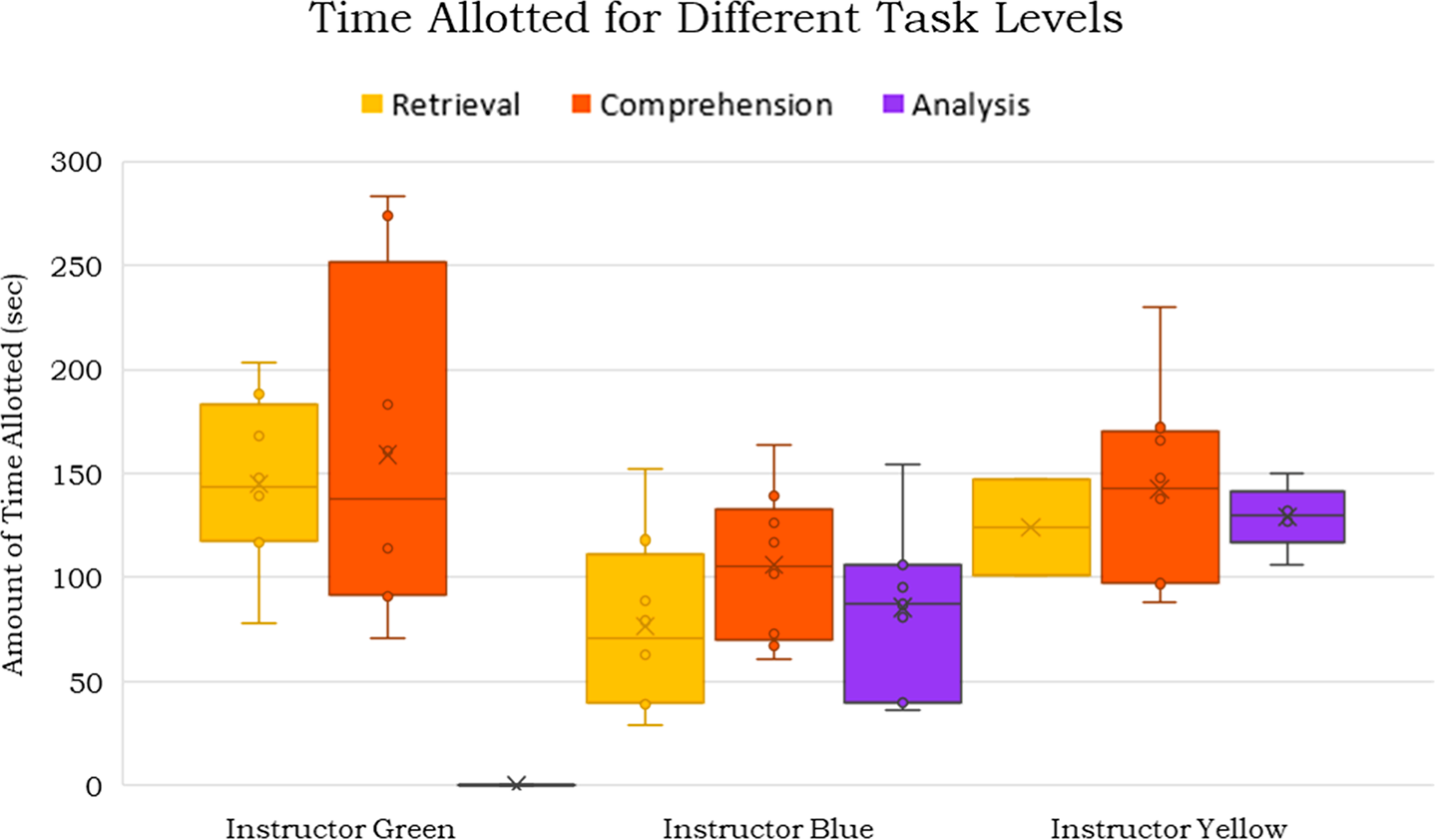
Box and whisker plot showing the distribution of the time allotted for questions at different task levels.

Because a large difference was seen in the student engagement between instructors during semester one, we wanted to take a closer look at the effect of different acts of facilitation on student engagement. These interruptions of Instructor Blue led to an overall decrease in the amount of time they allotted for students to work because they spent more time interrupting students’ working in groups than Instructor Green and Instructor Yellow. Because time seemed to play a factor in student engagement, we first looked at the average amount of time allotted for each question level for each instructor, shown in Figure 5.

Because higher-level questions, such as analysis, ask students to engage in more cognitively complex questions than lower-level questions like retrieval, it would be reasonable to assume that more time should be allotted for analysis level questions. What was observed across all instructors was that on average no additional time was given to higher-level questions. Instructor Green did have a wider distribution of time allotted for comprehension questions, but overall, the same amount of time was given for questions despite the difference in cognitive complexity of the questions. This may have set an expectation for engagement that implied there was only a certain amount of time for a question, no matter how complex, and that students should not need to spend extra time engaging deeply with a higher-level question. When students were unable to answer the higher-level question in the same amount of time as retrieval questions, it would not affect their grade, as points were based on participation rather than accuracy. This messaging could have set a condition for engagement that it does not matter what answer is input, so they do not need to be engaged for all questions.

**Figure 6:**
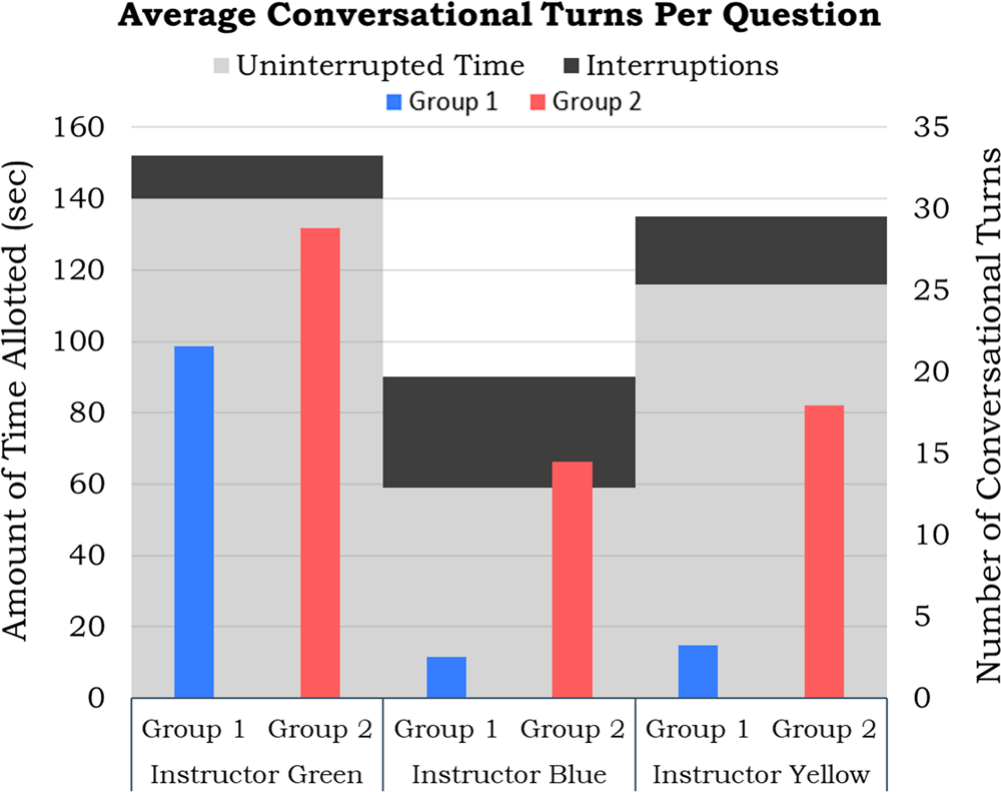
An average number of conversational turns and the average amount of time allotted for questions separated by the instructor from semester one.

Looking at the instructor level, in Figure 5 we can see that, on average, Instructor Blue offered the least amount of time for all question levels and Instructor Green offered the most time on average for questions. We wanted to investigate the effect that this variable average amount of time for questions had on student engagement, so we compared the average amount of time allotted for questions with the conversational turns of student groups, which can be seen in Figure 6.

From Figure 6, it can be seen that when observations began while Instructor Green was teaching, both student groups had approximately 25 conversational turns when allotted 140 s on average to answer questions. When Instructor Blue took over teaching, we see a big drop in the average amount of time allotted for questions with groups now only receiving 60 s to solve problems. This was followed by a decrease in the number of conversational turns for both groups. However, when normalizing for the variable amount of time allotted, group two was interacting with the same frequency given the time allotted with approximately 0.2 conversational turns per second throughout the semester. Group one’s average amount of conversational turns dropped from 0.15 conversational turns per second during the time Instructor Green taught to 0.04 conversational turns during the time that Instructor Blue taught. Consistent with the recovery of student social and cognitive engagement seen when Instructor Yellow took over teaching, there is an increase in the amount of time allotted for questions again with an uninterrupted average near 120 s, and we see a slight increase in the number of conversational turns for group one. However, this recovery was not seen in the number of conversational turns per second. This data shows that Instructor Blue did not allow sufficient time for students to answer problems; as a result, students in group one were less engaged during instruction.

These findings also highlight that differences in student engagement may not be able to be completely mediated by instructors. Despite the differences observed in instruction that could serve to demotivate students to interact, group two’s average number of conversational turns per second remained consistent. The members of group two may have been more disposed for active engagement than group one to explain their consistent amount of interaction, despite variations in the time allotted for questions. However, even though students in group two may have talked the same amount for all instructors, the group did engage in more individualistic activity and less knowledge application during the time that Instructor Blue was teaching, as seen in the analysis of social processing and knowledge dynamic seen above.

**Figure 7:**
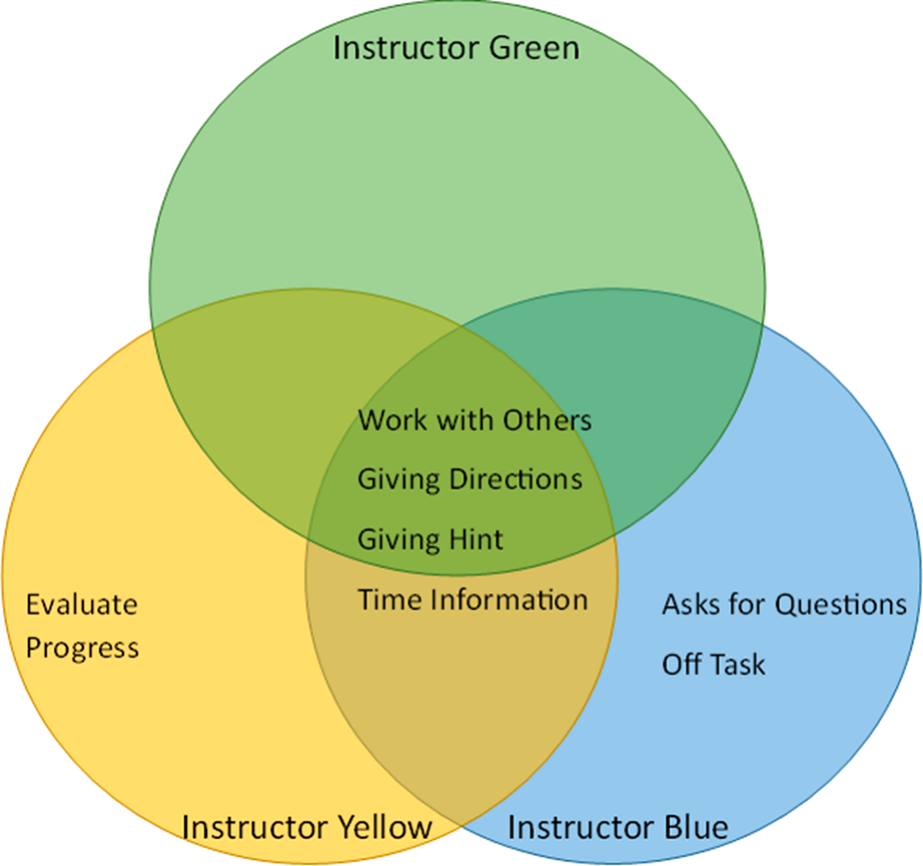
Venn Diagram showing the common content of instructor interaction codes seen across whole class instructor interruptions during question time for each instructor in semester one.

Although it seems the amount of time spent interrupting students influenced engagement, not all interruptions from an instructor are disruptive, as often instructors will intervene when students are stuck on a problem and need a more knowledgeable other to help guide them. A Venn diagram of the nature of instructor interruptions during whole close interruptions in the during-question section of facilitating questions can be seen in Figure 7.

Most of the interruptions given by all three instructors were interactions that can be helpful for students while they solve problems. All three instructors interrupted to encourage students to work with others, give directions or hints, or provide timely information related to the question. The only interruption that would be seen as nonhelpful would be the off-question interruption that was exhibited by Instructor Blue. Because all instructors were engaging in interruptions that are helpful for students’ progress, it appears that the increase in the frequency of interruptions seen for Instructor Blue likely led to the decrease in group engagement.

## Limitations

Because of the small number of groups and instructors observed at a large research-intensive midwestern university in the United States, this study may not represent other classrooms that are not held in a stadium-style lecture hall. The authors would also like to acknowledge that demographic information was not collected for any participants but recognize that participants may not reflect a diverse set of backgrounds that may be seen in other classrooms. Lastly, this study was completed based on student interaction during clicker questions, and if instructors use alternate forms of group activities, the interactions may differ.

## Implications

### Practice

Our results indicate that student engagement is likely to vary when different instructors teach. Despite efforts to keep instruction the same by asking clicker questions of similar cognitive complexity, variance in instruction can occur. Our results show that similar amounts of time allotted for all levels of questions may promote less engagement at higher cognitive levels. Instructors in introductory courses should take care to allow more time for students to solve problems that are more complex and be clear with their introduction of analysis level questions that students should be thinking deeply about the problem. We saw across semesters that when students were having answers explained interactively, that engagement was more collaborative, and groups engaged with knowledge more deeply. Instructors should try to facilitate clicker questions by placing the focus on the student, in-line with student-centered teaching approaches, and deliver questions dialogically, considering and discussing student ideas when able. Dialogic delivery of questions will let students share their thinking and become a partner in their learning rather than simply listening to an authoritative instructor. Instructors who are team teaching large enrollment courses should aim to facilitate questions similarly while paying attention to the communicative approach they are taking when explaining the answer to try and promote consistent social and cognitive engagement.

### Research

This study investigated the time allotted for questions and the communicative approaches of instructors as drivers for engagement to examine the effect that facilitation had on student engagement. Additional studies could be completed that look at other drivers for engagement such as student motivation. Our study did not ask students about their dispositions for engagement, but studies could be completed that interviewed students to gain additional information about motivators for engagement. We broadly examined student social and cognitive engagement in the aggregate, but future studies could take a closer look at student discourse to investigate student rapport and interpersonal communication that may also affect student disposition for engagement. Our data revealed that, across both semesters, the primary mode of facilitation was authoritative, consistent with the instructor-centered teaching method of didactic lecturing. Future studies could work with instructors to implement more dialogical practices into the facilitation of questions to determine the extent to which they promote positive student engagement consistently across groups and varying instructors. Additional studies could focus on identifying key features of highly effective teaching that can provide tangible implications for changing instructional practice. These potential pathways for research would be able to provide further insights into the complex ecosystem of large enrollment introductory chemistry courses in stadium-style classrooms.

## Conclusions

Student interactions and facilitation strategies in an introductory chemistry course were examined across two semesters of implementation to investigate the role of facilitation in supporting student engagement. Across both semesters, the nature of instruction appears to influence student drivers for acts of social and cognitive engagement. In semester one, during Instructor Blue’s time teaching, both groups engaged less interactively with an increase in individualistic and noninteractive social processing seen. Both groups’ engagement somewhat recovered when Instructor Yellow began teaching, but the recovery was much higher for group two. Seeing this difference in engagement with Instructor Blue led us to look at the trends of facilitation that may have acted as drivers for engagement within our setting.

Overall, instructors were positioning themselves as an authority on knowledge in the classroom by leading questions authoritatively similar to instructor-focused didactic lecturing. This means that even though students were engaging in active learning using “clicker” questions, the larger focus placed on the instructors may not have set up productive drivers for student engagement. This appeared to cause student groups to be less socially and cognitively engaged across the semesters. It makes sense that the students would develop expectations that the “clicker” questions were for personal practice and not a time for them to work their ideas out collaboratively when the instructor would just explain the answer in a minute. Additionally, the “clicker” questions were only graded based upon participation, so a student would need to be predisposed for active engagement to want to consistently interact. The only dramatic shift observed in student engagement occurred during semester one when Instructor Blue was teaching. This was due to a large percentage of interruptions that occurred during question sections, resulting in the least amount of time for students to work on problems in their groups. These results highlight the complexity of the classroom ecosystem related to student interactions and the role that facilitation plays in social and cognitive engagement. Further research is needed to evaluate other conditions and drivers for student acts of engagement in large enrollment introductory chemistry courses.

## Supporting Information Available

Additional coding results: Figures S1–S8 (PDF)
Additional coding results: Figures S1–S8 (DOCX)
Code definitions and facilitation map keys (PDF)
Code definitions and facilitation map keys (DOCX)

## References

[ref1] Bonwell C. C.; Eison J. A. Active Learning: Creating Excitement in the Classroom. 1991 ASHE-ERIC Higher Education Reports; ERIC Clearinghouse on Higher Education, The George Washington University: Washington, DC, 1991. https://eric.ed.gov/?id=ED336049 (accessed 2023-02-14).

[ref2] Cooper K. M.; Schinske J. N.; Tanner K. D. Reconsidering the Share of a Think-Pair-Share: Emerging Limitations, Alternatives, and Opportunities for Research. CBE Life Sci. Educ. 2021, 20 (1), fe1 10.1187/cbe.20-08-0200. 33444105 PMC8108495

[ref3] Deslauriers L.; McCarty L. S.; Miller K.; Callaghan K.; Kestin G. Measuring Actual Learning versus Feeling of Learning in Response to Being Actively Engaged in the Classroom. Proc. Natl. Acad. Sci. U. S. A. 2019, 116 (39), 19251–19257. 10.1073/pnas.1821936116. 31484770 PMC6765278

[ref4] Duncan D. Clickers: A New Teaching Aid with Exceptional Promise. Astron. Educ. Rev. 2006, 5, 70–88. 10.3847/AER2006005.

[ref5] Freeman S.; Eddy S. L.; McDonough M.; Smith M. K.; Okoroafor N.; Jordt H.; Wenderoth M. P. Active Learning Increases Student Performance in Science, Engineering, and Mathematics. Proc. Natl. Acad. Sci. U. S. A. 2014, 111 (23), 8410–8415. 10.1073/pnas.1319030111. 24821756 PMC4060654

[ref6] Theobald E. J.; Hill M. J.; Tran E.; Agrawal S.; Arroyo E. N.; Behling S.; Chambwe N.; Cintrón D. L.; Cooper J. D.; Dunster G.; Grummer J. A.; Hennessey K.; Hsiao J.; Iranon N.; Jones L.; Jordt H.; Keller M.; Lacey M. E.; Littlefield C. E.; Lowe A.; Newman S.; Okolo V.; Olroyd S.; Peecook B. R.; Pickett S. B.; Slager D. L.; Caviedes-Solis I. W.; Stanchak K. E.; Sundaravardan V.; Valdebenito C.; Williams C. R.; Zinsli K.; Freeman S. Active Learning Narrows Achievement Gaps for Underrepresented Students in Undergraduate Science, Technology, Engineering, and Math. Proc. Natl. Acad. Sci. U. S. A. 2020, 117 (12), 6476–6483. 10.1073/pnas.1916903117. 32152114 PMC7104254

[ref7] Atieh E. L.; York D. M. Give and Take: Narrowing the Gap between Theory and Practice of Peer Instructors over Time. J. Chem. Educ. 2022, 99 (10), 3370–3385. 10.1021/acs.jchemed.2c00170.

[ref8] Cole R. S. Sustaining the Adoption of Active Learning. In Active Learning in the Analytical Chemistry Curriculum; ACS Symposium Series; American Chemical Society: 2022; Vol. 1409, pp 297–306. 10.1021/bk-2022-1409.ch016.

[ref9] Henderson C.; Dancy M. H. Barriers to the Use of Research-Based Instructional Strategies: The Influence of Both Individual and Situational Characteristics. Phys. Rev. Spec. Top. - Phys. Educ. Res. 2007, 3 (2), 020102 10.1103/PhysRevSTPER.3.020102.

[ref10] Lund T. J.; Stains M. The Importance of Context: An Exploration of Factors Influencing the Adoption of Student-Centered Teaching among Chemistry, Biology, and Physics Faculty. Int. J. STEM Educ. 2015, 2 (1), 13 10.1186/s40594-015-0026-8.

[ref11] Shadle S.; Marker A.; Earl B. Faculty Drivers and Barriers: Laying the Groundwork for Undergraduate STEM Education Reform in Academic Departments. Int. J. STEM Educ. 2017, 4, 8 10.1186/s40594-017-0062-7. 30631664 PMC6310369

[ref12] Andrews T. M.; Leonard M. J.; Colgrove C. A.; Kalinowski S. T. Active Learning Not Associated with Student Learning in a Random Sample of College Biology Courses. CBE Life Sci. Educ. 2011, 10 (4), 394–405. 10.1187/cbe.11-07-0061. 22135373 PMC3228657

[ref13] Stains M.; Vickrey T. Fidelity of Implementation: An Overlooked Yet Critical Construct to Establish Effectiveness of Evidence-Based Instructional Practices. CBE Life Sci. Educ. 2017, 16 (1), rm1 10.1187/cbe.16-03-0113. 28213585 PMC5332058

[ref14] Kranzfelder P.; Lo A. T.; Melloy M. P.; Walker L. E.; Warfa A.-R. M. Instructional Practices in Reformed Undergraduate STEM Learning Environments: A Study of Instructor and Student Behaviors in Biology Courses. Int. J. Sci. Educ. 2019, 41 (14), 1944–1961. 10.1080/09500693.2019.1649503.

[ref15] Hattie J. Visible Learning: A Synthesis of Over 800 Meta-Analyses Relating to Achievement. Routledge & CRC Press. https://www.routledge.com/Visible-Learning-A-Synthesis-of-Over-800-Meta-Analyses-Relating-to-Achievement/Hattie/p/book/9780415476188 (accessed 2023-03-03).

[ref16] Scherr R. E.; Hammer D. Student Behavior and Epistemological Framing: Examples from Collaborative Active-Learning Activities in Physics. Cogn. Instr. 2009, 27 (2), 147–174. 10.1080/07370000902797379.

[ref17] Young K. K.; Talanquer V. Effect of Different Types of Small-Group Activities on Students’ Conversations. J. Chem. Educ. 2013, 90 (9), 1123–1129. 10.1021/ed400049a.

[ref18] Haak D. C.; HilleRisLambers J.; Pitre E.; Freeman S. Increased Structure and Active Learning Reduce the Achievement Gap in Introductory Biology. Science 2011, 332 (6034), 1213–1216. 10.1126/science.1204820. 21636776

[ref19] Reinholz D. L.; Shah N. Equity Analytics: A Methodological Approach for Quantifying Participation Patterns in Mathematics Classroom Discourse. J. Res. Math. Educ. 2018, 49 (2), 140–177. 10.5951/jresematheduc.49.2.0140.

[ref20] Reinholz D. L.; Bradfield K.; Apkarian N. Using Analytics to Support Instructor Reflection on Student Participation in a Discourse-Focused Undergraduate Mathematics Classroom. Int. J. Res. Undergrad. Math. Educ. 2019, 5 (1), 56–74. 10.1007/s40753-019-00084-7.

[ref21] Neill C.; Cotner S.; Driessen M.; Ballen C. J. Structured Learning Environments Are Required to Promote Equitable Participation. Chem. Educ. Res. Pract. 2019, 20 (1), 197–203. 10.1039/C8RP00169C.

[ref22] Shekhar P.; Borrego M. ‘Not Hard to Sway’: A Case Study of Student Engagement in Two Large Engineering Classes. Eur. J. Eng. Educ. 2018, 43, 585–596. 10.1080/03043797.2016.1209463.

[ref23] Mason D.; Verdel E. Gateway to Success for At-Risk Students in a Large-Group Introductory Chemistry Class. J. Chem. Educ. 2001, 78 (2), 252 10.1021/ed078p252.

[ref24] Cooper K. M.; Downing V. R.; Brownell S. E. The Influence of Active Learning Practices on Student Anxiety in Large-Enrollment College Science Classrooms. Int. J. STEM Educ. 2018, 5 (1), 23 10.1186/s40594-018-0123-6. 30631713 PMC6310416

[ref25] Aina J. K.; Sunday O. S.; Ayinde G. I. Teachers’ Effectiveness and Its Influence on Students’ Learning. Adv. Soc. Sci. Res. J. 2015, 2 ( (4), ). 10.14738/assrj.24.1082.

[ref26] Brooks D. C. Space Matters: The Impact of Formal Learning Environments on Student Learning. Br. J. Educ. Technol. 2011, 42 (5), 719–726. 10.1111/j.1467-8535.2010.01098.x.

[ref27] Cotner S.; Loper J.; Walker J. D.; Brooks D. C. It’s Not You, It’s the Room” - Are the High-Tech, Active Learning Classrooms Worth It?. J. Coll. Sci. Teach. 2013, 42 (6), 82–88. 10.2505/4/jcst13_042_06_82.

[ref28] Bernstein D. A. Does Active Learning Work? A Good Question, but Not the Right One. Scholarsh. Teach. Learn. Psychol. 2018, 4 (4), 290–307. 10.1037/stl0000124.

[ref29] Dosch M. V. The Course Fit Us: Differentiated Instruction in the College Classroom. Ph.D., The University of North Dakota. http://www.proquest.com/docview/1024340982/abstract/46CD4FCCE9944F55PQ/1 (accessed 2022-11-10).

[ref30] Beatty I. D.; Gerace W. J.; Leonard W. J.; Dufresne R. J. Designing Effective Questions for Classroom Response System Teaching. Am. J. Phys. 2006, 74 (1), 31–39. 10.1119/1.2121753.

[ref31] Judson E.; Sawada D. Learning from Past and Present: Electronic Response Systems in College Lecture Halls. J. Comput. Math. Sci. Teach. 2002, 21 (2), 167–181.

[ref32] Lombardi D.; Shipley T. F.; Bailey J. M.; Bretones P. S.; Prather E. E.; Ballen C. J.; Knight J. K.; Smith M. K.; Stowe R. L.; Cooper M. M.; Prince M.; Atit K.; Uttal D. H.; LaDue N. D.; McNeal P. M.; Ryker K.; St. John K.; van der Hoeven Kraft K. J.; Docktor J. L. The Curious Construct of Active Learning. Psychol. Sci. Public Interest 2021, 22 (1), 8–43. 10.1177/1529100620973974.

[ref33] Lawson M. A.; Lawson H. A. New Conceptual Frameworks for Student Engagement Research, Policy, and Practice. Rev. Educ. Res. 2013, 83 (3), 432–479. 10.3102/0034654313480891.

[ref34] The New Taxonomy of Educational Objectives, 2nd ed.; Marzano R. J., Kendall J. S., Eds.; Corwin: Thousand Oaks, CA, 2006.

[ref35] McHugh M. L. Interrater Reliability: The Kappa Statistic. Biochem. Medica 2012, 22 (3), 276–282. 10.11613/BM.2012.031. PMC3900052 23092060

[ref36] MAXQDA | All-In-One Qualitative & Mixed Methods Data Analysis Tool. MAXQDA. https://www.maxqda.com/ (accessed 2022-12-08).

[ref37] Scott P. H.; Mortimer E. F.; Aguiar O. G. The Tension between Authoritative and Dialogic Discourse: A Fundamental Characteristic of Meaning Making Interactions in High School Science Lessons. Sci. Educ. 2006, 90 (4), 605–631. 10.1002/sce.20131.

[ref38] Merriam S. B. author. Qualitative Research: A Guide to Design and Implementation/Sharan B. Merriam, Elizabeth J. Tisdell., 4th ed.; Jossey-Bass Higher and Adult Education Series; Jossey-Bass, a Wiley brand: San Francisco, CA, 2016.

[ref39] Reid J. W.; Kirbulut Gunes Z. D.; Fateh S.; Fatima A.; Macrie-Shuck M.; Nennig H. T.; Quintanilla F.; States N. E.; Syed A.; Cole R.; Rushton G. T.; Shah L.; Talanquer V. Investigating Patterns of Student Engagement during Collaborative Activities in Undergraduate Chemistry Courses. Chem. Educ. Res. Pract. 2022, 23 (1), 173–188. 10.1039/D1RP00227A.

