# Supplementary material for: Let Students Work: Analysis of the Role of Differing Facilitation on Student Engagement in a Large Stadium-Style Lecture Hall
Source: J Chem Educ. 2023 Nov 1;100(11):4237–48. doi: 10.1021/acs.jchemed.3c00750 (PMC10653220; doi:10.1021/acs.jchemed.3c00750)
Supplement: Supplementary file 1 — ed3c00750_si_001.pdf [file ed3c00750_si_001.pdf]

Supporting Information

Let students work: Analysis of the role of differing facilitation on student engagement in a large stadium-style lecture hall

Nicole E. States ^, Carson Lovig, Karsten Martin, Hannah T. Nennig, and Renée S. Cole*

Department of Chemistry, University of Iowa, Iowa City, IA, 52242

^Now at Saint Louis University, St. Louis, MO, 63103

Corresponding author email: renee-cole@uiowa.edu

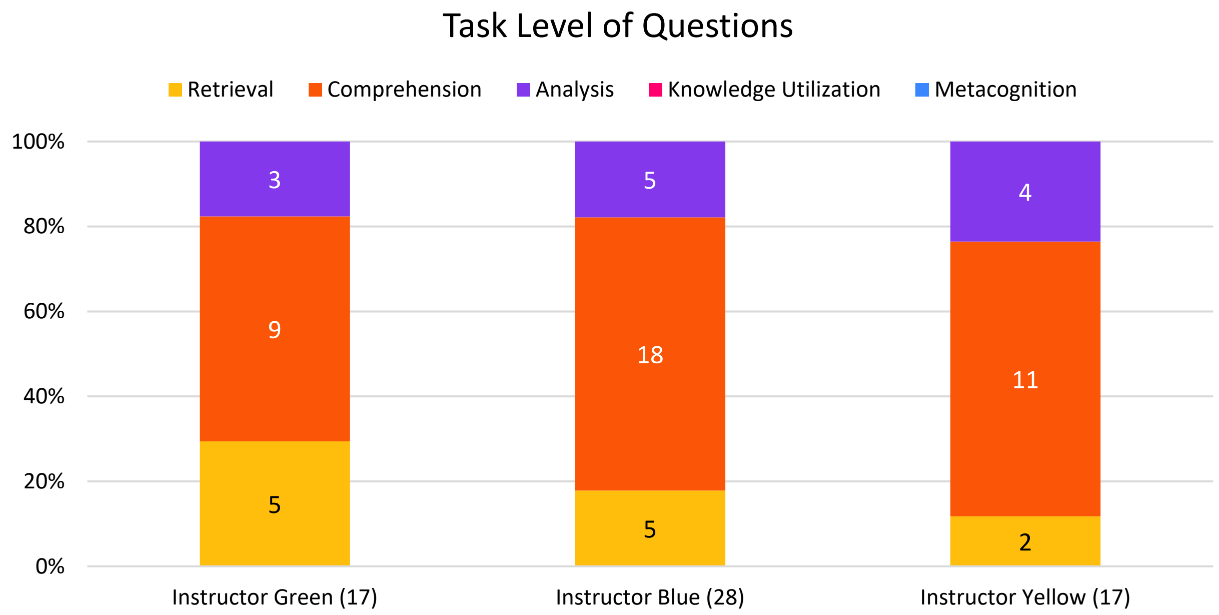

Figure S1. Percentage of each task level across instructors during semester one

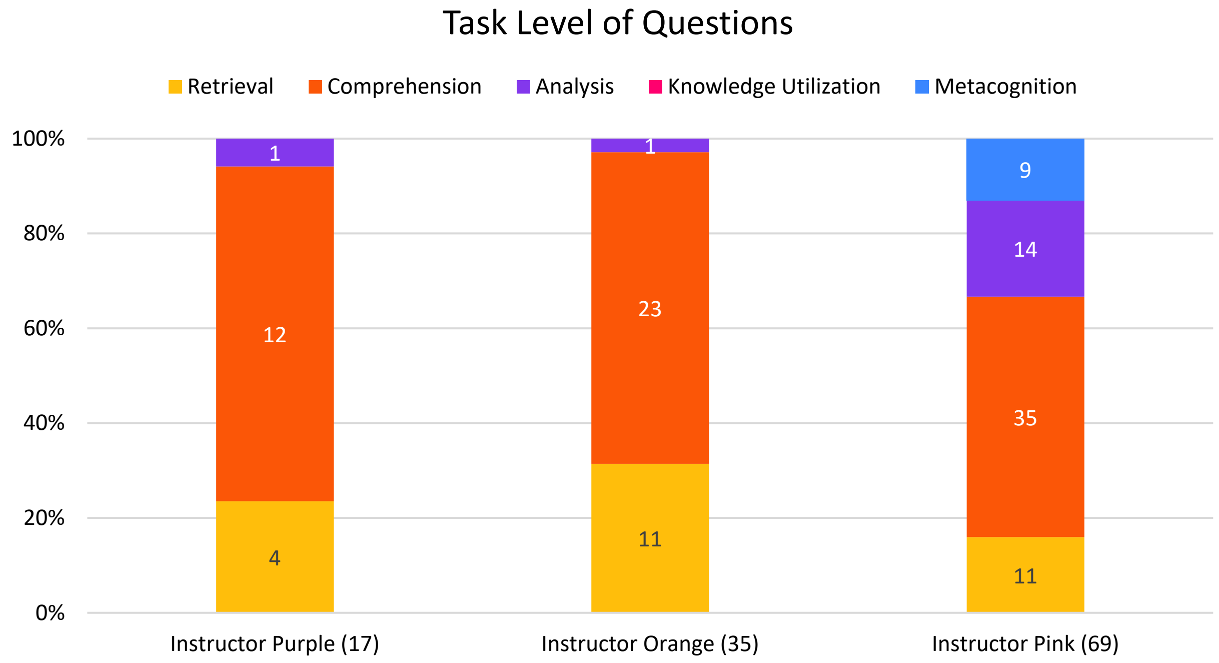

Figure S2. Percentage of each task level across instructors during semester two

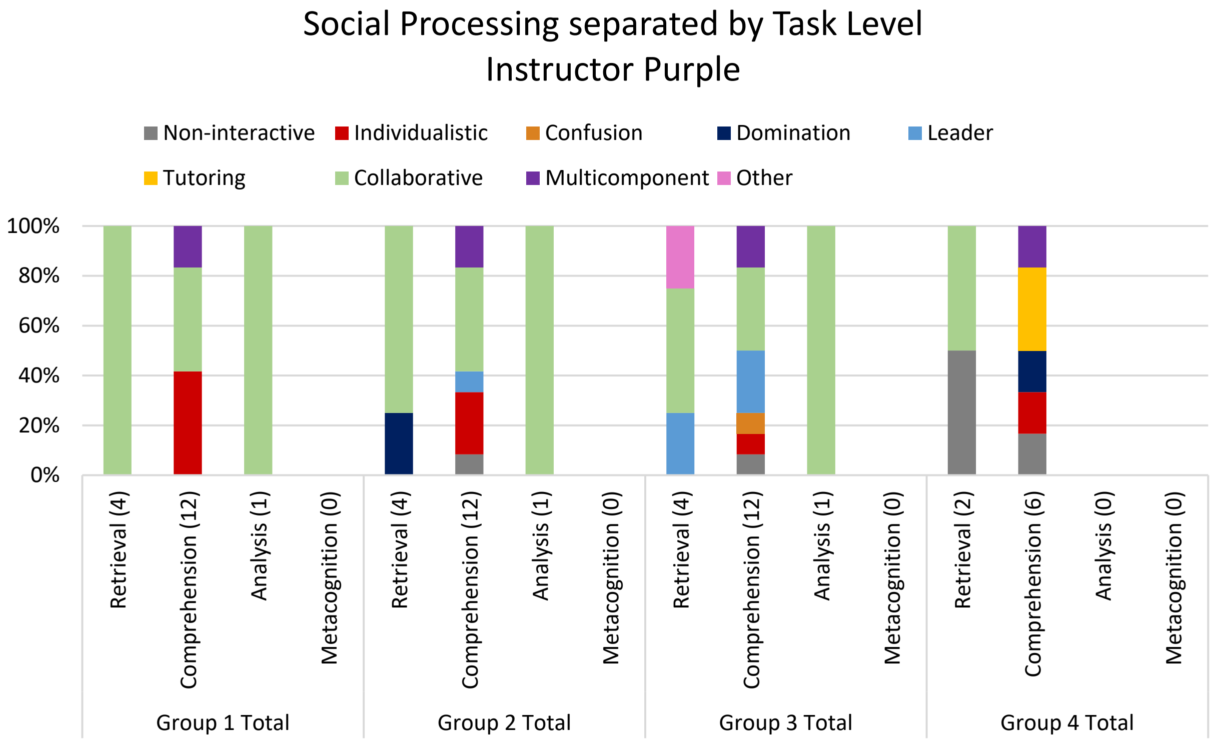

Figure S3. Percentages of each social processing across student groups during semester two separated by task level for Instructor Purple

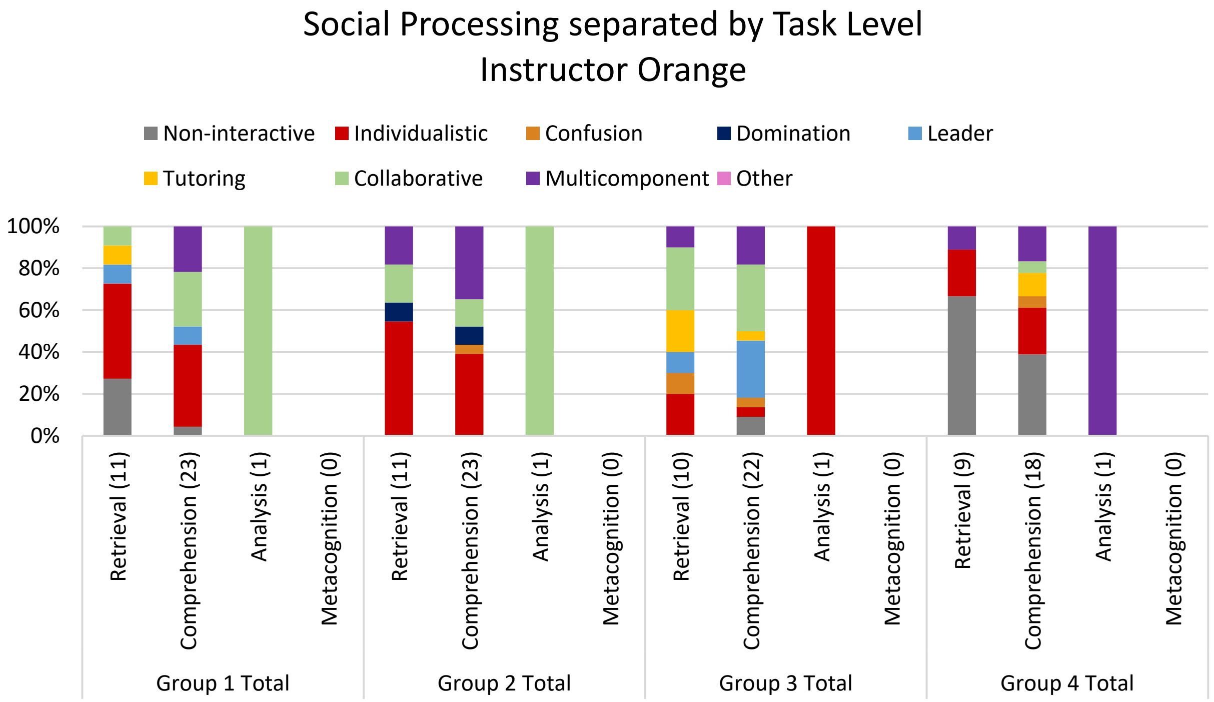

Figure S4. Percentages of each social processing across student groups during semester two separated by task level for Instructor Orange

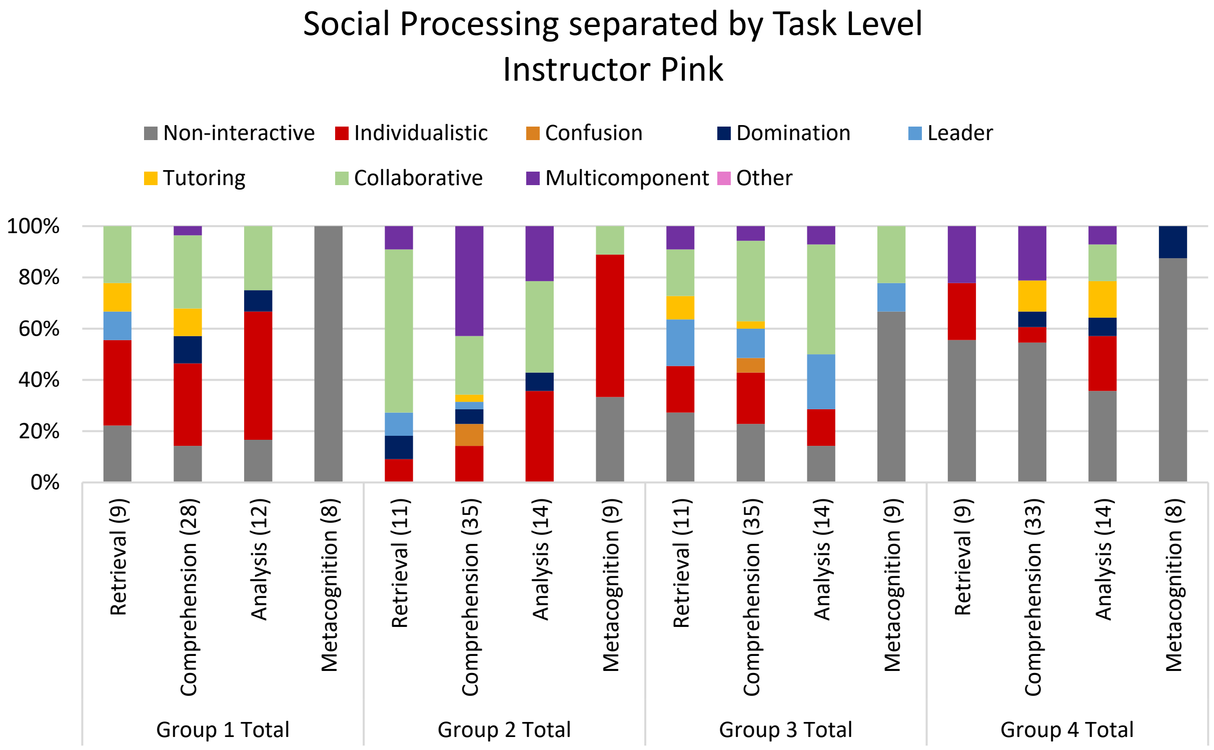

Figure S5. Percentages of each social processing across student groups during semester two separated by task level for Instructor Pink

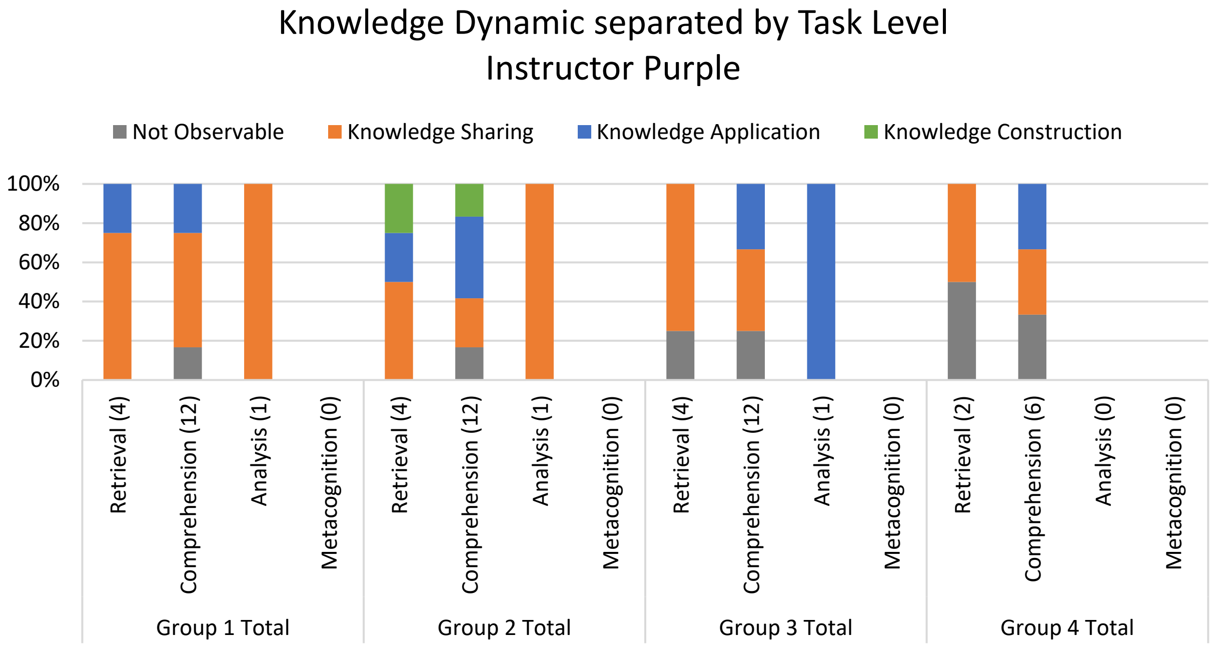

Figure S6. Percentages of each knowledge dynamic across student groups during semester two separated by task level for Instructor Purple

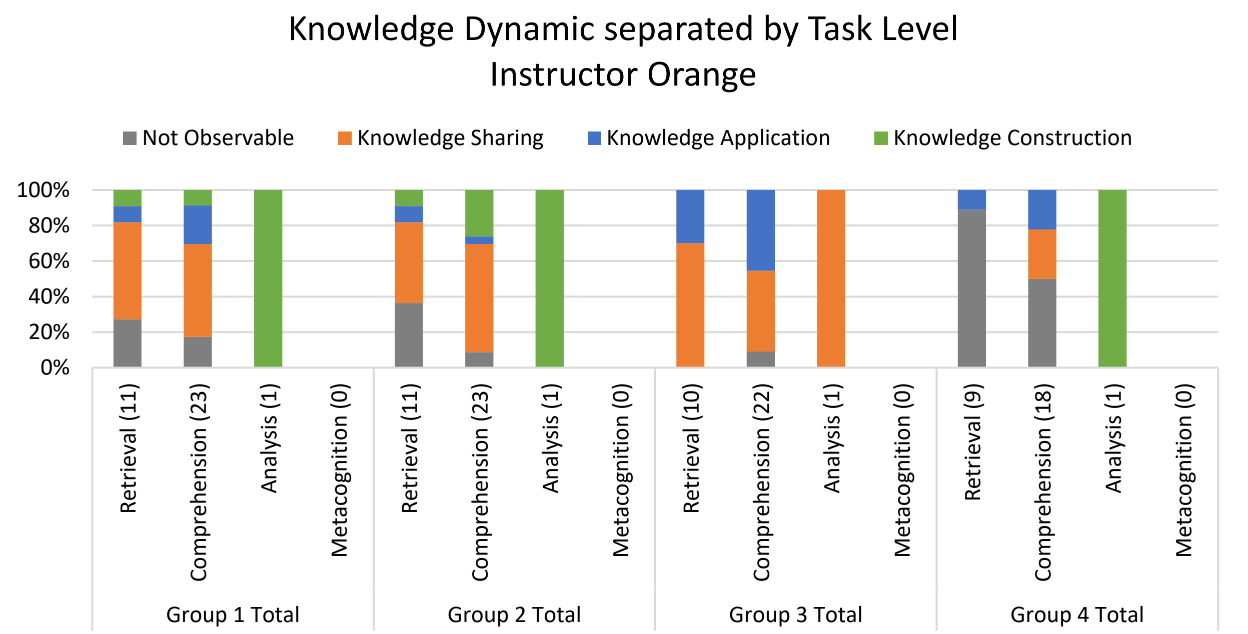

Figure S7. Percentages of each knowledge dynamic across student groups during semester two separated by task level for Instructor Orange

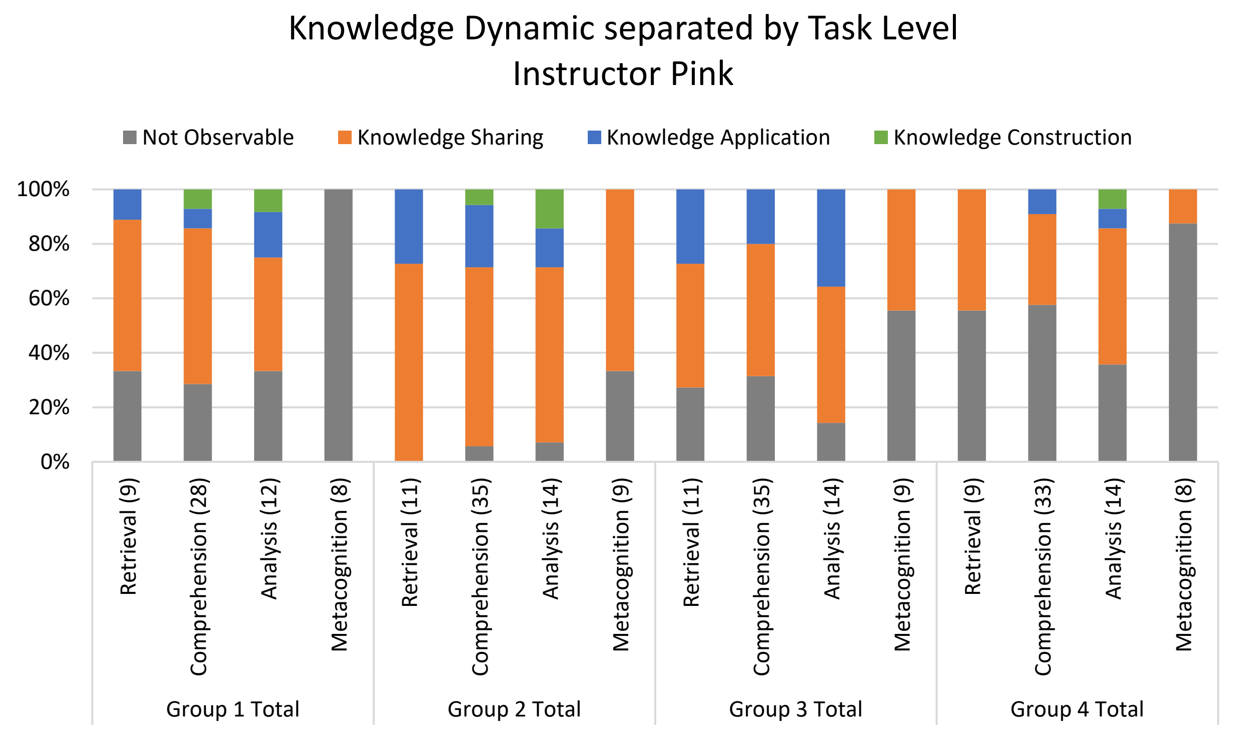

Figure S8. Percentages of each knowledge dynamic across student groups during semester two separated by task level for Instructor Pink
