# Supplementary material for: Let Students Work: Analysis of the Role of Differing Facilitation on Student Engagement in a Large Stadium-Style Lecture Hall
Source: J Chem Educ. 2023 Nov 1;100(11):4237–48. doi: 10.1021/acs.jchemed.3c00750 (PMC10653220; doi:10.1021/acs.jchemed.3c00750)
Supplement: Supplementary file 3 — ed3c00750_si_003.pdf [file ed3c00750_si_003.pdf]

Supporting Information

Let students work: Analysis of the role of differing facilitation on student engagement in a large stadium-style lecture hall

Nicole E. States ^, Carson Lovig, Karsten Martin, Hannah T. Nennig, and Renée S. Cole*

Department of Chemistry, University of Iowa, Iowa City, IA, 52242

^Now at Saint Louis University, St. Louis, MO, 63103

Corresponding author email: renee-cole@uiowa.edu

Table S1. Code definitions for social processing interactions. Adapted from Reid et al. 2022[1]

Social Processing	Definitions
Collaborative	Students are co-constructing ideas and generating products together
Confusion	Students are too confused to really generate the expected product or make confident progress for a question
Domination	One student constructs the response for the group while not considering, ignoring, or rejecting input given by others
Leader	One student primarily constructs the response due to a lack of contribution from others
Tutoring	One or more students ask questions that another student, “tutor”, responds to. This is either done by the tutor guiding the students, “tutees”, through the problem asking for their ideas or just by the tutor explaining their reasoning without asking input from the tutees who asked the question.
Individualistic	Students are working independently and are not having conversations about the question products
Non-interactive	Students are not having any conversation, but there is no proof of individualistic work

Table S2. Code definitions used for knowledge dynamic interactions. Adapted from Reid et al. 2022[1].

Knowledge Dynamic	Definition
Not Applicable	No knowledge dynamic is seen due to a lack of student interaction with knowledge. Inclusive of when students just check in with only the final answer.
Knowledge Sharing	The focus of the group interactions is based on sharing information to answer the task without questioning the why/how of the utterances presented
Knowledge Application	The focus of the group interactions is based on applying a formula/method/concept and relating that to an understanding of how it relates to the explanation of solving the problem
Knowledge Construction	The focus of group interactions is based on sharing information and building upon the ideas of others by questioning or critiquing the why/how of the ideas presented

Table S3. Key for facilitation video coding visualization scheme

Code	Shape	Color
Closing Task	Border	#ff9900
Introduction of Task	Border	#00BFFF
During Task	Border	#00cc00

Communicative Approach<br>Interactive Authoritative	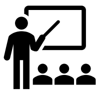	NA
Communicative Approach<br>Noninteractive Authoritative	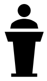	NA
Communicative Approach<br>Interactive Dialogical	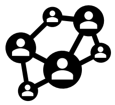	NA
Communicative Approach<br>Noninteractive Dialogical	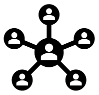	NA
Managing<br>Announcing Question Period	square	#0000cc
Managing<br>Call on Student	square	#0000FF
Managing<br>Closing Class	square	#98CBFE
Managing<br>Classroom Management	square	#3CCDCC
Managing<br>Closing Question Period	square	#00ffff
Managing<br>Course Reminders	square	#b3ffb3
Managing<br>Encouragement	square	#006666
Managing<br>Encouraging Collaboration	square	#6601CB
Managing<br>Opening Class Period	square	#b3b3ff
Managing<br>Overview	square	#00cc00
Managing<br>Giving Directions	square	#9965FF
Managing<br>Reading Prompt	square	#660066
Managing<br>Reconsider Answer	square	#666699
Managing<br>Time Information	square	#000066
Managing<br>Study	square	#000066
Questioning<br>Ask Content Question (Unanswered)	triangle	#F832CC

Questioning<br>Asks Content Question (Answered)	triangle	#990699
Questioning<br>Asks for Questions	triangle	#ff0066
Questioning<br>Asks for Whole Class Response (Content)	triangle	#F72B66
Questioning<br>Asks for Whole Class Response (Non-content)	triangle	#FBCC99
Questioning<br>Asks Non-content Question (Answered)	triangle	#F99934
Questioning<br>Asks Non-content Question (Unanswered)	triangle	#9A1900
Questioning<br>Cold Call Asks Question	triangle	#991900
Questioning<br>Evaluate Progress	triangle	#993366
Questioning Rhetorical Asks for Questions	triangle	#F76601
Relaying<br>Answer Student Question	circle	#00cc00
Relaying<br>Answer Assessment	circle	#ffff00
Relaying<br>Explains Answer	circle	#00cc00
Relaying<br>Gives Analogy	circle	#146601
Relaying<br>Giving Hint	circle	#666634
Relaying<br>Provides Task Answer	circle	#99CC01
Relaying<br>Responds to Student Answer	circle	#CC9900

References

(1) Reid, J. W.; Gunes, Z. D. K.; Fateh, S.; Fatima, A.; Macrie-Shuck, M.; Nennig, H. T.; Quintanilla, F.; States, N. E.; Syed, A.; Cole, R.; Rushton, G. T.; Shah, L.; Talanquer, V. Investigating Patterns of Student Engagement during Collaborative Activities in Undergraduate Chemistry Courses. *Chem. Educ. Res. Pract.* **2022**, *23* (1), 173–188. https://doi.org/10.1039/D1RP00227A.
