# Supplementary material for: Let Students Work: Analysis of the Role of Differing Facilitation on Student Engagement in a Large Stadium-Style Lecture Hall
Source: J Chem Educ. 2023 Nov 1;100(11):4237–48. doi: 10.1021/acs.jchemed.3c00750 (PMC10653220; doi:10.1021/acs.jchemed.3c00750)
Supplement: Supplementary file 4 — ed3c00750_si_004.docx [file ed3c00750_si_004.docx]

Supporting Information

Let students work: Analysis of the role of differing facilitation on student engagement in a large stadium-style lecture hall

Nicole E. States ^, Carson Lovig, Karsten Martin, Hannah T. Nennig, and Renée S. Cole*

Department of Chemistry, University of Iowa, Iowa City, IA, 52242

^Now at Saint Louis University, St. Louis, MO, 63103

Corresponding author email: renee-cole@uiowa.edu

Table S1. Code definitions for social processing interactions. Adapted from Reid et al. 2022^1^

| Social Processing | Definitions |
| --- | --- |
| Collaborative | Students are co-constructing ideas and generating products together |
| Confusion | Students are too confused to really generate the expected product or make confident progress for a question |
| Domination | One student constructs the response for the group while not considering, ignoring, or rejecting input given by others |
| Leader | One student primarily constructs the response due to a lack of contribution from others |
| Tutoring | One or more students ask questions that another student, “tutor”, responds to. This is either done by the tutor guiding the students, “tutees”, through the problem asking for their ideas or just by the tutor explaining their reasoning without asking input from the tutees who asked the question. |
| Individualistic | Students are working independently and are not having conversations about the question products |
| Non-interactive | Students are not having any conversation, but there is no proof of individualistic work |

Table S2. Code definitions used for knowledge dynamic interactions. Adapted from Reid et al. 2022^1^.

| Knowledge Dynamic | Definition |
| --- | --- |
| Not Applicable | No knowledge dynamic is seen due to a lack of student interaction with knowledge. Inclusive of when students just check in with only the final answer. |
| Knowledge Sharing | The focus of the group interactions is based on sharing information to answer the task without questioning the why/how of the utterances presented |
| Knowledge Application | The focus of the group interactions is based on applying a formula/method/concept and relating that to an understanding of how it relates to the explanation of solving the problem |
| Knowledge Construction | The focus of group interactions is based on sharing information and building upon the ideas of others by questioning or critiquing the why/how of the ideas presented |

Table S3. Key for facilitation video coding visualization scheme

| Code | Shape | Color |
| --- | --- | --- |
| Closing Task | Border | #ff9900 |
| Introduction of Task | Border | #00BFFF |
| During Task | Border | #00cc00 |
| Communicative Approach  Interactive Authoritative | 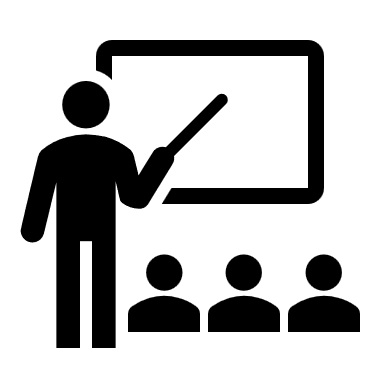 | NA |
| Communicative Approach  Noninteractive Authoritative | 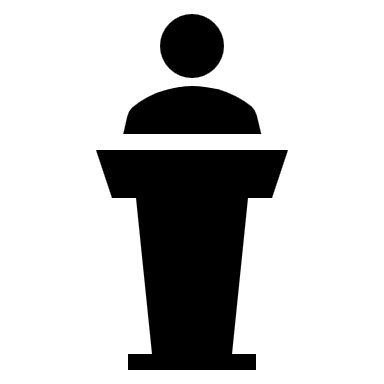 | NA |
| Communicative Approach  Interactive Dialogical | 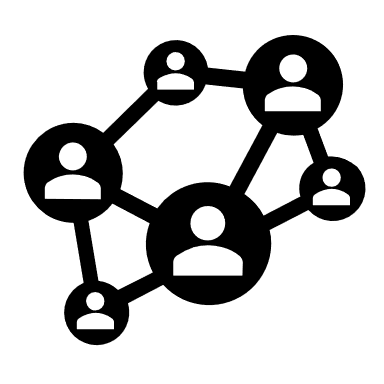 | NA |
| Communicative Approach  Noninteractive Dialogical | 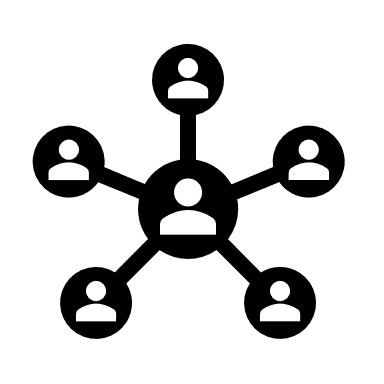 | NA |
| Managing  Announcing Question Period | square | #0000cc |
| Managing  Call on Student | square | #0000FF |
| Managing  Closing Class | square | #98CBFE |
| Managing  Classroom Management | square | #3CCDCC |
| Managing  Closing Question Period | square | #00ffff |
| Managing  Course Reminders | square | #b3ffb3 |
| Managing  Encouragement | square | #006666 |
| Managing  Encouraging Collaboration | square | #6601CB |
| Managing  Opening Class Period | square | #b3b3ff |
| Managing  Overview | square | #00cc00 |
| Managing  Giving Directions | square | #9965FF |
| Managing  Reading Prompt | square | #660066 |
| Managing  Reconsider Answer | square | #666699 |
| Managing  Time Information | square | #000066 |
| Managing  Study | square | #000066 |
| Questioning  Ask Content Question (Unanswered) | triangle | #F832CC |
| Questioning  Asks Content Question (Answered) | triangle | #990699 |
| Questioning  Asks for Questions | triangle | #ff0066 |
| Questioning  Asks for Whole Class Response (Content) | triangle | #F72B66 |
| Questioning  Asks for Whole Class Response (Non-content) | triangle | #FBCC99 |
| Questioning  Asks Non-content Question (Answered) | triangle | #F99934 |
| Questioning  Asks Non-content Question (Unanswered) | triangle | #9A1900 |
| Questioning  Cold Call Asks Question | triangle | #991900 |
| Questioning  Evaluate Progress | triangle | #993366 |
| Questioning Rhetorical Asks for Questions | triangle | #F76601 |
| Relaying  Answer Student Question | circle | #00cc00 |
| Relaying  Answer Assessment | circle | #ffff00 |
| Relaying  Explains Answer | circle | #00cc00 |
| Relaying  Gives Analogy | circle | #146601 |
| Relaying  Giving Hint | circle | #666634 |
| Relaying  Provides Task Answer | circle | #99CC01 |
| Relaying  Responds to Student Answer | circle | #CC9900 |

References

(1) Reid, J. W.; Gunes, Z. D. K.; Fateh, S.; Fatima, A.; Macrie-Shuck, M.; Nennig, H. T.; Quintanilla, F.; States, N. E.; Syed, A.; Cole, R.; Rushton, G. T.; Shah, L.; Talanquer, V. Investigating Patterns of Student Engagement during Collaborative Activities in Undergraduate Chemistry Courses. *Chem. Educ. Res. Pract.* **2022**, *23* (1), 173–188. https://doi.org/10.1039/D1RP00227A.
